# Right in two: capabilities of ion mobility spectrometry for untargeted metabolomics

**DOI:** 10.3389/fmolb.2023.1230282

**Published:** 2023-08-04

**Authors:** Tessa Moses, Karl Burgess

**Affiliations:** ^1^ EdinOmics, RRID:SCR_021838, University of Edinburgh, Max Born Crescent, Edinburgh, United Kingdom; ^2^ Institute of Quantitative Biology, Biochemistry and Biotechnology, School of Biological Sciences, University of Edinburgh, Edinburgh, United Kingdom

**Keywords:** chromatography, separation, drift time, drift tube, travelling wave, trapped, high-field asymmetric

## Abstract

This mini review focuses on the opportunities provided by current and emerging separation techniques for mass spectrometry metabolomics. The purpose of separation technologies in metabolomics is primarily to reduce complexity of the heterogeneous systems studied, and to provide concentration enrichment by increasing sensitivity towards the quantification of low abundance metabolites. For this reason, a wide variety of separation systems, from column chemistries to solvent compositions and multidimensional separations, have been applied in the field. Multidimensional separations are a common method in both proteomics applications and gas chromatography mass spectrometry, allowing orthogonal separations to further reduce analytical complexity and expand peak capacity. These applications contribute to exponential increases in run times concomitant with first dimension fractionation followed by second dimension separations. Multidimensional liquid chromatography to increase peak capacity in metabolomics, when compared to the potential of running additional samples or replicates and increasing statistical confidence, mean that uptake of these methods has been minimal. In contrast, in the last 15 years there have been significant advances in the resolution and sensitivity of ion mobility spectrometry, to the point where high-resolution separation of analytes based on their collision cross section approaches chromatographic separation, with minimal loss in sensitivity. Additionally, ion mobility separations can be performed on a chromatographic timescale with little reduction in instrument duty cycle. In this review, we compare ion mobility separation to liquid chromatographic separation, highlight the history of the use of ion mobility separations in metabolomics, outline the current state-of-the-art in the field, and discuss the future outlook of the technology. “Where there is one, you’re bound to divide it. Right in two”, James Maynard Keenan.

## 1 Introduction

Global untargeted metabolomics aims at facilitating our understanding of the dynamics of the chemical composition of biological systems. Metabolites in heterogenous systems are chemically diverse and vary in their abundances, with many endogenous metabolites existing at very low concentrations. The primary goal of metabolomics is the unbiased relative quantification of each metabolite in a biological system. For comprehensive metabolite coverage of biological systems to be successful, multiple separation techniques are needed.

In this review, we outline the principles of separation systems as applied to mass spectrometry based metabolomics. We focus on the capabilities of the increasingly commonly used technique of ion mobility, hyphenated (or not) to liquid chromatography. We evaluate the current state-of-the-art of ion mobility metabolomics in its various guises and provide a forward-looking summary of where we can expect the technology to move in future.

## 2 Liquid chromatography

The term liquid chromatography was originally described by Michael Tswett in 1906 (Tswett, 1968). The method initially used to separate plant pigments (hence the name) has now become a powerful and ubiquitous technique routinely applied to separate and purify complex mixtures of molecules. Briefly, analytes are dissolved in a solvent and then passed through a column filled with a stationary phase. The stationary phase can be made of various materials, such as silica or polymer beads, which are packed uniformly into the column. As the analytes pass through the column, different molecules interact with the stationary phase, depending on their physicochemical properties. This interaction causes the molecules to separate and travel through the column at different rates, creating peaks.

There are many different stationary phases, allowing separations to be tailored depending on the particular characteristics of the analytes in question. A detailed analysis of stationary phases is beyond the scope of this article, and there are already many excellent reviews on the subject (Zou et al., 2002; Zhang, 2008; West et al., 2010; Sobańska, 2021), but the most commonly applicable phases in metabolomics in general are reversed phase (RP, which separates on the basis of hydrophobicity) and hydrophilic interaction liquid chromatography (HILIC, which separates on the basis of hydrophilicity).

### 2.1 Advantages of liquid chromatography separation

While there have been many excellent studies in the metabolomics discipline conducted using direct infusion mass spectrometry (Maleki et al., 2018; Dou et al., 2023; Sun et al., 2023; Wolthuis et al., 2023) and flow injection mass spectrometry (Zang et al., 2018), chromatographic separations are commonly hyphenated to mass spectrometry. This coupling adds a further dimension of separation, beyond mass spectrometry itself, which in essence is a separation.

The main aim of applying chromatographic separations up-front of a mass spectral analysis is to reduce the complexity of highly complex biological samples, in an effort to ameliorate the effects of dynamic range (Want et al., 2005) and ion suppression (Furey et al., 2013). Furthermore, chromatography provides a very valuable concentration of each analyte as it leaves the column as a peak, substantially improving its signal-to-noise ratio and making it more likely to be detected and quantified, as opposed to direct infusion mass spectrometry.

### 2.2 Limitations of liquid chromatography for global metabolomics

One of the main limitations of chromatography is its lack of reproducibility and its heavy reliance on a few common stationary phases. Reversed-phase chromatography, for example, is now the most well-developed and ubiquitous separation medium in analytical chromatography, and rather than use a different column, it is common to use ion-pairing reagents to adapt the chemistry of an analyte to a reversed-phase system (Gong, 2015). Unfortunately, the vast majority of ion-pairing reagents are not mass spectrometry compatible, comprising (as many do) inorganic salts or highly ionizable organic compounds in millimolar concentrations that overwhelm the detection of less abundant endogenous compounds in the system being studied. Various powerful metabolomics methods exploit these properties to perform more targeted analyses, but for broad-based untargeted metabolomics, analysts are typically limited to RP or HILIC with volatile organic acids, bases and salts used as separation systems. Thus, a major limitation of liquid chromatography, as applied to mass spectrometry is that the separation gradient is responsible for much of the selectivity of the system, which can lead to situations where global separations are poorly suited to the separation of closely related compounds.

## 3 Ion mobility spectrometry

Ion mobility spectrometry (IMS), also known as gaseous electrophoresis, plasma chromatography (Revercomb and Mason, 1975), or ion chromatography (Helden et al., 1995), is an electrophoretic technique that separates ions based on their mobility in gas phase when subjected to an electric field. Traditional IMS measurements determine the drift velocities of gaseous ions in a weak electric field at a constant temperature. The applied electrical field accelerates ions through the drift region, which is counteracted by the drift gas that impedes ion progress. The speed of ion movement or drift velocity (ν_d_) is therefore proportional to the strength of the applied electric field (E), with mobility (K) of the ion being the constant of proportionality. The mobility of an ion is determined by its shape, size, and charge in the given drift gas. As a result, ions traverse the drift region at a velocity that is proportional to the inverse of their collision cross section (CCS), a physical property that reflects the geometric shape of the ion in the specific gas (Borsdorf and Eiceman, 2006). More compact structural conformations undergo fewer collisions with the drift gas and therefore have smaller CCS values, than extended planar structures. Thus, the CCS feature provides insight into the overall shape of the molecule, while ion mobility allows rapid separation of mixtures, including isobaric ions, typically within milliseconds.

### 3.1 Types of ion mobility spectrometry

**FIGURE 1:**
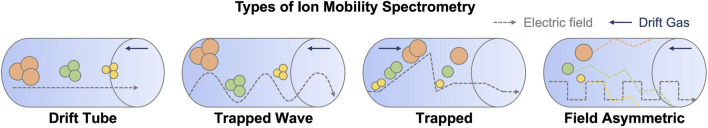
Schematic representation of the different types of ion mobility and the principles of separation. The direction of drift gas flow and electric field are indicated with dark blue and grey arrows, respectively.

There are four major types of ion mobility spectrometry (Figure 1). The most common and oldest ion mobility analysers consist of a drift tube containing an inert gas, typically dubbed ‘drift tube ion mobility spectrometry or DTIMS’ (Kirk et al., 2019), as described above. Travelling wave ion mobility spectrometry (TWIMS) is a more recent modification of the traditional drift tube on similar hardware, with the addition of an electrostatic pulse waveform that propagates along the tube, allowing nonlinear resolution of ions at the expense of ion heating (Shvartsburg and Smith, 2008). Ion heating is one of the more significant limitations of TWIMS as ion temperatures can reach 551–774 K (Merenbloom et al., 2012) and can lead to dissociation in small molecules (Morsa et al., 2011). More recent developments in this technology include structures for lossless ion manipulation (SLIM) and cyclic ion mobility (Giles et al., 2019). The SLIM architecture consists of a sandwich structure, with parallel, mirror image electrode arrays that can be configured for either conventional or travelling wave ion mobility (Ibrahim et al., 2017). Currently, available commercial instruments based on the technology configure the system in a serpentine array, significantly extending the ion mobility path length to a total of 13 m utilizing 44 U-turns. Resolving power varies depending on the configuration of the ion optics and the voltages applied, but resolutions of around 300 are available (May et al., 2021). The cyclic IMS instrument incorporates a doughnut-shaped cyclic ion mobility (cIM) device that allows ions to be separated via a user-designated number of repetitive 98 cm paths. The drawback to the instrument is that faster, lighter ions eventually end up ‘lapping’ slower ones, and to this end the instrument brackets the cIM with ion traps, allowing isolation and selection of pre-and post-separation ions. This allows some unprecedented and complex ion mobility experiments, such as the one described by (Sisley et al., 2020), to be performed.

Another type of ion mobility analyser is high-field asymmetric ion mobility spectrometry (FAIMS). This relies on the use of a changing compensation voltage as a filter across a gas flow counter to the direction of the ions. FAIMS was originally published by Buryakov et al. (1993), and has since found its way into several commercial instruments. It is generally employed as a filter (Canterbury et al., 2008), rather than a separation device and has therefore been sparsely used in liquid chromatography coupled to ion mobility multidimensional separations.

Recently, the Park group introduced the trapped ion mobility spectrometry (TIMS) device (Ridgeway et al., 2016). Unlike the previous systems, in TIMS the ions are held in a trapping device and exposed to a moving column of gas, based typically on a modification of ion funnel technology. This trap and release substantially reduce the dimensions of the ion mobility device. Ions are separated based on their physicochemical properties as in conventional drift tube ion mobility, however, the movement of the gas and ions are reversed. In TIMS, ramping the electric field gradient (E) releases ions in descending order of their mobility (K). For an excellent review of TIMS, see (Ridgeway et al., 2018). Parallel accumulation, serial fragmentation (PASEF) is an experimental methodology available on TIMS instruments that uses the unique capabilities of the ion mobility trapping device to accumulate ions, and then sequentially pulse them into a quadrupole for isolation and fragmentation (Meier et al., 2018). Thus, in a single drift spectrum, fragment patterns for each MSMS spectrum are resolved via ion mobility. This dramatically increases the acquisition rate of data dependent acquisition experiments (Meier et al., 2015). It has recently been modified to support data-independent acquisition (dia-PASEF) for proteomics applications (Demichev et al., 2022), which provides an intriguing possibility for improving metabolomics analysis.

### 3.2 Advantages of ion mobility as a separation technology

Regardless of the specific type of ion mobility device, there are two main advantages of ion mobility spectrometry. Firstly, it allows the separation of isomeric analytes. As discussed in Section 2.2, one of the major limitations of chromatography is that while an optimal liquid chromatography gradient can separate closely related ions, the generally applicable rapid gradients used with untargeted analysis often preclude the separation of isomeric compounds with closely related structures. While this is an issue for chromatography, modern ion mobility instruments with resolutions greater than 100 can now separate many isomeric ions within milliseconds. By coupling ion mobility to chromatography, multidimensional separation can be performed, allowing unprecedented resolution of complex mixtures without extending separation times or employing complex fractionation strategies.

Similar to the first, the second advantage of ion mobility comes from the modern high-resolution IM instruments. Collisional cross section is a fundamental property of an ion, and it can therefore be calculated with greater accuracy than predicting retention times on columns. While ion mobility devices measure ‘drift times’ rather than CCS directly, all modern instruments can calculate CCS from drift times, and this directly addresses one of the biggest challenges in metabolomics—the unambiguous identification of metabolites.

This latter poses an important challenge for the application of IMS to metabolomics. CCSs are able to be determined from experimental parameters via the Mason-Schamp equation (Siems et al., 2012) in DTIMS instruments, while for other types of IMS, calibration standards with known CCS values (derived from DTIMS experiments) must be used (Dodds and Baker, 2019). In practice, even on DTIMS instruments, a typical use for metabolomics, e.g., single field multiplexed analysis, require calibration standards derived from slower, more complex stepped-field methodologies (Dodds and Baker, 2019). Due to the development of large-scale, experimentally determined libraries (Zheng et al., 2017; Picache et al., 2019), as well as appropriate calibration, CCS features provide an additional criterion for metabolite annotation.

### 3.3 Limitations of ion mobility

Sensitivity has been the perennial issue with ion mobility mass spectrometry. In standard ion mobility analysis, analytes are introduced to the ion mobility device as a packet, followed by ion separation and concomitant detection in the mass spectrometer. Before the next packet of ions can be introduced to the ion mobility device, the slowest ion in the current packet must pass through and exit the IM device. This means that during ion mobility separation, all ions that would otherwise be analysed are lost. Furthermore, due to imperfect electronics, ions of interest can also be lost during the ion mobility separation itself. There have been many attempts to improve sensitivity, such as improving overall instrument design as outlined in 3.1 (Deng et al., 2016), advances in the construction of the instrument with improved lens designs and electric field generation, and the use of multiplexing. Multiplexing is a technique used to decrease ion packet losses in ion mobility mass spectrometry. Multiple ion packets are pulsed into a single ion mobility device in a pseudo-random order. This creates a patterning effect in the ion mobility analysis that can be deconvoluted by the use of a sliding window algorithm during data analysis. This technique has been reported to reduce sensitivity losses from 99% to 50% (Reinecke et al., 2019).

Current, commercially available ion mobility instruments possess resolving power in the hundreds, compared to the tens of thousands of theoretical plates typical in chromatography (for a fascinating discussion on the relationship between resolution parameters for chromatography and spectrometry see (Rokushika et al., 1985)). Consequently, reproducibility is a key parameter to confidently assign collisional cross sections to analytes. Interlaboratory studies have been performed, demonstrating RSDs of 0.14% and ∼1.5% in TWIMS-based devices (Hernández-Mesa et al., 2020; Righetti et al., 2020) and 0.29% for DTIMS-based devices (Stow et al., 2017), which compares favourably to chromatography retention time RSD of 5%–20% depending on analyte (Madji Hounoum et al., 2015).

A further limitation to ion mobility is that it does not get around the problem of matrix effect, a somewhat complex term in ‘omics disciplines, particularly metabolomics. Typically, the “matrix” corresponds to all of the components of a sample one does not want to analyse, while the “analytes” are the components one wants to detect. While all sample preparation methodologies are biased, a good ‘targeted’ analysis aims to strip out the vast majority of the “matrix” while leaving the “analytes” in place. In untargeted metabolomics one does not have this luxury, and the “matrix” and the analytes are (apart from contaminants, buffers, salts, etc.) mostly the same thing. The interaction between these contaminants, as well as the more abundant analytes within the ion mobility device can result in ion suppression (or ion enhancement).

Ion suppression is commonly associated with electrospray ionization, although matrix effects and suppression of ionization are also found in electron ionization used with gas chromatography (Yarita et al., 2015) and nanoelectrospray ionization (Kourtchev et al., 2020). Ion suppression occurs in the ion source, but matrix effects such as ion-ion interactions and space charging can occur in trapping instruments (Hohenester et al., 2020). Thus, ion mobility separations, occurring as they do, after ionization, provide no reduction on in-source ion suppression. Consequently, suppression resulting from, flow injection or direct infusion mass spectrometry will not be ameliorated by the use of IMS, and could result in additional artifacts in measurement (Levin et al., 2014).

## 4 Hyphenating ion mobility to mass spectrometry reduces sample complexity

As previously stated, the primary purpose of separation in mass spectrometry studies is the reduction in sample complexity and reduction of matrix effects. The human metabolome database currently contains 217,920 compounds (Wishart et al., 2022), demonstrating the scale of metabolomics researchers’ analytical challenge. Of these, 8,369 metabolites are currently listed as ‘detected’ in human tissues, with the remainder either predicted or derived from other sources, such as the microbiome and the diet. With the impact of ion suppression (see 3.3) and the limited dynamic range of mass spectrometers (Want et al., 2005), it is very common to perform some separation of analytes before they are presented to the mass spectrometer.

An exception to this is mass spectrometry imaging (MSI), a technique where mass spectrometry is used to raster across a tissue or other spatially resolved sample, providing a spectrum per pixel. Individual ions can be collated to generate images of the distribution of a molecular species to, for example, a tissue structure or feature. Of course, a typical matrix-assisted laser desorption ionization (MALDI) (Tanaka et al., 1988) or desorption electrospray ionization (DESI) (Takáts et al., 2004) process is extremely challenging to hyphenate to chromatography as extraction of analytes and ionization are performed directly from the sample. Therefore, MSI sees considerable benefits from the incorporation of ion mobility separation prior to mass spectrometry (Spraggins et al., 2019), regardless of any impact of matrix effects, as high-resolution IMS can separate isobaric compounds without prior chromatographic separation, while also maintaining acquisition speed.

Outside the world of MSI, hyphenation of chromatography to mass spectrometry has been used to ameliorate ion suppression and the effects of limited dynamic range for decades, and the recent advances in ion mobility peak capacities have made this a viable addition, or alternative, to chromatography separations.

## 5 The use of multidimensional separation and best practices

Typical multidimensional separation workflows consist of coupling multiple liquid chromatography-based separation chemistries and hyphenating to mass spectrometry. A full overview of metabolomics applications with liquid chromatography is beyond the scope of this article. For a comprehensive review of recent methods please read (Lv et al., 2019).

Many examples of ion mobility used as a separation method for metabolomics studies appear in the literature (see Table 1). Of the 28 tabulated articles, nine perform no prior separation to analysis via ion mobility-mass spectrometry. Of these, four are single-cell studies using a variety of methods (laser ablation electrospray ionization, matrix-assisted laser desorption ionization, microsampling), where the addition of chromatography would be challenging, if not impossible. For the remainder, a variety of chromatography methods are hyphenated. The most common remains the use of RP chromatography employing C18 columns, but a growing number of HILIC chromatography methods are beginning to be applied. A single method by Causon et al. incorporates heart-cutting two-dimensional liquid chromatography into a metabolomics workflow, while an additional two methods incorporate both HILIC and RP chromatography, performed separately, into their analyses.

**TABLE 1:** Typical workflows in liquid chromatography ion mobility mass spectrometry utilizing multidimensional separation principles.

Type of ion mobility	Instrument used	Separation pre-ion mobility	Run length (min)	Sample	Comments	Reference
TWIMS	Waters	Metabolites: BEH Amide	30	Not listed		Paglia and Astarita (2017)
	Synapt G2S	Lipids: C18				
DTIMS	Agilent	Waters Atlantis HSS T3 RPLC	21	*Pichia pastoris*		Feuerstein et al. (2021)
	6560					
TIMS	Bruker timsTOF fleX	Matrix-assisted laser desorption ionization (MALDI)	N/A	Human kidney	IM separation only, mass spectrometry imaging	Neumann et al. (2020)
DTIMS	Agilent	LC-MS: Atlantis T3 C18	20	*Pichia pastoris*	Combines conventional LC-IMMS and heart cutting 2D LC-IMMS	Causon et al. (2019)
	6560	Heart cutting: Hypercarb porous graphetised carbon				
DTIMS	Agilent	Capillary electrophoresis	Not listed	Mass Spectrometry Metabolite Library of Standards (Sigma)		Drouin et al. (2021)
	6560					
TWIMS	Waters	Flow injection	3	Serum (prostate cancer)	No chromatographic separation	Zang et al. (2018)
	Synapt G2-S					
DTIMS	Modified Agilent	Laser ablation electrospray ionization (LAESI)	Not listed	*Allium cepa*	IM separation only, single-cell mass spectrometry	Taylor et al. (2021)
	6538					
DTIMS	Agilent	Agilent Zorbax Eclipse Plus C18	22.5	Human serum (xenobiotics)		Foster et al. (2022)
	6560					
TWIMS	Waters	Waters HSS T3 RPLC	26	Human serum		Tebani et al. (2016)
	Synapt G2-S					
DTIMS	Modified Agilent	Waters BEH C18	5	*Pichia pastoris*	Data independent MS/MS	Mairinger et al. (2019)
	6560					
TIMS	Bruker timsTOF Pro	Bruker Solo C18	23	Olive oil		Drakopoulou et al. (2021)
TWIMS	Waters	Waters HSS T3	29	Ginseng root, leaf, bud		Li et al. (2021)
	Vion IM-QToF					
TWIMS	Waters	Waters CORTECS C18	18	Human plasma (orange metabolites)		Lacalle-Bergeron et al. (2020)
	Vion IM-QToF					
DTIMS	Agilent	Not listed	Not listed	Not listed	Protocol paper	Reisdorph et al. (2019)
	6560					
DTIMS	Modified Thermo	Direct infusion	Not listed	Standards and bovine heart extract		Maleki et al. (2018)
	LTQ-Velos					
DTIMS	Custom Ionwerks	Direct infusion	Not listed	Rat lymph		Kaplan et al. (2013b)
DTIMS	Tofwerk	Direct infusion	20	Rat brain tissue after cocaine administration		Kaplan et al. (2013a)
	Resistive Glass Ion Mobility-ToF MS					
TWIMS	Waters	Waters BEH Amide (HILIC) and Waters BEH C18 (RP)	HILIC: 12	Saposhnikoviae Radix		Wang et al. (2020)
	Vion IM-QToF		RP: 17			
FAIMS	Owlstone FAIMS with Agilent 6230	Agilent Poroshell 120 HILIC	13	Human urine		Szykuła et al. (2019)
TWIMS	Waters	Direct infusion	2	Exhaled human breath condensate (cystic fibrosis patients)		Zang et al. (2017)
	Synapt G2-S					
TWIMS	Waters	Phenomenex Kinetex C18 and CORTECS HILIC	18	Herbal cigarettes (14 herbs plus tobacco)		Gil-Solsona et al. (2021)
	Vion IM-QToF					
TWIMS	Waters	Inertsyl Phenyl-3	47	Zucker rat plasma (high fat diet)		Wickramasekara et al. (2013)
	Synapt G2-S					
TWIMS	Waters	Waters HSS T3 C18	30	Mouse feces (bile diversion surgery)		Poland et al. (2019)
	Synapt G2-S					
TWIMS	Waters	LAESI	Not listed	Human neuroblasts	IM separation only—single cell mass spectrometry	Stopka and Vertes (2019)
	Synapt G2-S					
FAIMS	Sciex	Single cell microsampling	Not listed	*Raphanus sativus* single cells	IM separation only—single cell mass spectrometry	Fujii et al. (2015)
	SelexION QTRAP 5500					
TWIMS	Waters	Waters HSS T3 C18	25	*Vangueria agrestis* glycosides and terpenoids		Avula et al. (2020)
	Vion IMS-QToF					
DTIMS	Agilent	Agilent HILIC-Z	4	*Bacillus* sp. Plant growth promoting rhizobacteria		Pičmanová et al. (2022)
	6560					
TWIMS	Waters	Waters BEH Amide	3.3	Rat urine (tienilic acid study)		King et al. (2019)
	Synapt G2-S					

Liquid chromatography hyphenated with ion mobility mass spectrometry has become a powerful technique for reducing complexity in untargeted metabolomics. Publications should, of course, adhere to the most appropriate rigorous standards for reporting, the most recent of which is currently (Kirwan et al., 2022), but ion mobility mass spectrometry is a relatively recent development and standards are still evolving. At minimum, the parameters for the ion mobility device should be recorded along with those for the mass spectrometer it is hyphenated to. This is relatively straightforward for dedicated hybrid instruments, but can be more complex for aftermarket or less intrinsically-linked hardware. Additionally, calibration standards should be noted and the parameters of the calibration should be recorded. Typically these are performed before every batch, but IM calibration samples at both the beginning and the end of a run would provide good evidence that the system was working to appropriate parameters during sample acquisition. CCS libraries, both predicted (Zhou et al., 2020) and experimentally determined (Nichols et al., 2018) are now commonly used in ion mobility mass spectrometry analyses. Both are useful but which library and what criteria (typically a 1%–2% window is used to match CCS) were used is critically important information in the quality assessment of identifications, and should be rigorously reported.

Of course, libraries are not valuable without software to support their use, and academic software that supports the diversity of ion mobility datasets has been relatively slow in development in comparison to the advances in instrumentation. The three most commonly used packages in the field are MS-Dial (Tsugawa et al., 2015), Skyline (MacLean et al., 2010) and MzMine (Schmid et al., 2023). All three are graphical applications that provide peak picking, metabolite annotation and statistics, with Skyline being more closely aimed at targeted applications and MS-Dial having the benefit of integrated large-scale libraries. Ideally, the development of modular tools accessible via R and/or Python would support rapid advancement in terms of new features, that could then be backported into more attractive GUI applications while allowing the possibility of building pipelines into interfaces such as Workflow4Metabolomics (Giacomoni et al., 2015).

## 6 Conclusion and prospects

Ion mobility spectrometry hyphenated to mass spectrometry has seen rapid technological advancement in the previous 10 years. Initially confined to a few specialists, it is finding itself now routinely applied in proteomics and metabolomics studies for the valuable additional information it provides. Given its capacity for extremely rapid separations, we anticipate a great future for the technology and expect to see further developments to improvements in resolution for the technology.
